# Current variables, definitions and endpoints of the European Cardiovascular Magnetic Resonance Registry

**DOI:** 10.1186/1532-429X-11-43

**Published:** 2009-11-05

**Authors:** Anja Wagner, Oliver Bruder, Steffen Schneider, Detlev Nothnagel, Peter Buser, Guillem Pons-Lado, Thorsten Dill, Vinzenz Hombach, Massimo Lombardi, Albert C van Rossum, Juerg Schwitter, Jochen Senges, Georg V Sabin, Udo Sechtem, Heiko Mahrholdt, Eike Nagel

**Affiliations:** 1 Department of Cardiology, Hahnemann University Hospital, Drexel University College of Medicine, Philadelphia, USA; 2 Department of Cardiology and Angiology, Elisabeth Hospital, Essen, Germany; 3 Institut für Herzinfarktforschung, Ludwigshafen, Germany; 4 Department of Cardiology, Klinikum Ludwigsburg, Ludwigsburg, Germany; 5 Department of Cardiology, University Hospital Basel, Basel, Switzerland; 6 Cardiac Imaging Unit, Hospital de la Santa Creu I Sant Pau, Universitat Autonoma de Barcelona, Spain; 7 Department of Cardiology, Kerkhoff-Klinik, Bad Nauheim, Germany; 8 Department of Internal Medicine II, Cardiology, University of Ulm, Ulm, Germany; 9 Clinical Physiology Institute, CNR National Research Council, Pisa, Italy; 10 Department of Cardiology, VU Medical Centre, Amsterdam, The Netherlands; 11 Cardiac MR Centre, University Hospital Lausanne, Lausanne, Switzerland; 12 Department of Cardiology, Robert Bosch Medical Centre, Stuttgart, Germany; 13 Division of Imaging Sciences, King's College London BHF Centre of Excellence and NIHR Biomedical Research Centre, St Thomas' Trust, London, UK

## Abstract

**Background:**

Cardiovascular Magnetic Resonance (CMR) is increasingly used in daily clinical practice. However, little is known about its clinical utility such as image quality, safety and impact on patient management. In addition, there is limited information about the potential of CMR to acquire prognostic information.

**Methods:**

The European Cardiovascular Magnetic Resonance Registry (EuroCMR Registry) will consist of two parts: 1) Multicenter registry with consecutive enrolment of patients scanned in all participating European CMR centres using web based online case record forms. 2) Prospective clinical follow up of patients with suspected coronary artery disease (CAD) and hypertrophic cardiomyopathy (HCM) every 12 months after enrolment to assess prognostic data.

**Conclusion:**

The EuroCMR Registry offers an opportunity to provide information about the clinical utility of routine CMR in a large number of cases and a diverse population. Furthermore it has the potential to gather information about the prognostic value of CMR in specific patient populations.

## Introduction

Cardiovascular disease is the major cause of mortality and morbidity in the United States and in Europe. Cardiac imaging is an integral part of the evaluation and management of patients with known or suspected cardiovascular disease. Echocardiography and single photon emission computed tomography are well-established imaging techniques to diagnose cardiovascular diseases and to provide prognostic information. Cardiovascular Magnetic Resonance (CMR) is also established in many sites, but its clinical value regarding impact on decision-making and patient safety is not well understood. Like traditional techniques, CMR can assess cardiac anatomy, quantify ventricular and valvular function, as well as identify areas of necrosis, scarring and ischemia. There is an increasing body of evidence that CMR is superior to traditional imaging techniques due to the fact that it offers a higher spatial resolution and the unique ability of tissue characterization [1-3]. Consequently, the use of CMR in daily clinical routine has rapidly grown in recent years. However, there is very limited knowledge of its clinical utility, impact and safety. The EuroCMR registry provides the unique opportunity to acquire data on the prognostic impact of CMR studies performed in a real-life scenario. To address questions of clinical utility, impact, safety and prognostic value, the EuroCMR Registry consists of two parts: 1) A documentation of image quality, safety, and impact on patient management in a large number of cases to assess the clinical utility of CMR in daily clinical routine. 2) A prospective clinical follow up of patients with suspected coronary artery (CAD) disease and hypertrophic cardiomyopathy (HCM) to assess prognostic data.

## Methods

### Study design

The EuroCMR Registry consists of two parts: 1) Multicenter registry with consecutive enrolment of patients scanned in all participating CMR centres. Indications for CMR had to be according to the SCMR consensus appropriateness criteria for CMR [4], and all procedures must be performed in compliance with the standardised SCMR recommended protocols. 2) Prospective clinical follow up of patients with suspected coronary artery disease and hypertrophic cardiomyopathy every 12 months after enrolment to assess prognostic data. The study is to be approved by local institutional review boards and all patients will be asked for informed consent.

### Study population

1) Multicenter registry: The registry will include consecutive patients who undergo CMR in one of the participating sites. The only exclusion criteria are contraindications for CMR.

2) Prospective clinical follow up: Participating centres are required to enrol consecutive patients in at least one of the specific protocols. Inclusion criteria for the two specific protocols are as follows:

A) Suspected-CAD: Patients with suspected coronary artery disease undergoing a combined CMR protocol including evaluation of left ventricular function, assessment of myocardial ischemia by adenosine stress perfusion or high-dose dobutamine CMR, as well as detection of myocardial infarction using late gadolinium enhanced CMR.

B) HCM-SCD: Patients with hypertrophic cardiomyopathy undergoing a combined CMR protocol including left ventricular function, rest perfusion, and detection of myocardial scarring by late gadolinium enhanced CMR. The diagnosis of hypertrophic cardiomyopathy is based on the demonstration of a hypertrophied, non-dilated left ventricle (wall thickness of at least 15 mm in adults or the equivalent relative to body-surface area in children) in the absence of another cardiac or systemic disease capable of producing a similar degree of hypertrophy. In adult relatives of the patients with hypertrophic cardiomyopathy, a wall thickness of 13 mm or more will be considered a criterion for diagnosis.

Additional exclusion criteria for the two specific protocols are as follows:

A) Suspected-CAD: Absence of patient informed consent, known CAD by invasive coronary angiography or previous myocardial infarction.

B) HCM-SCD: Absence of patient informed consent, known CAD by invasive coronary angiography or previous myocardial infarction, left ventricular hypertrophy of other causes (e.g. hypertension, valvular heart disease).

In addition, the EuroCMR registry is open to new specific protocols to be added and operated by interested researchers. Thus, the investigators of the EuroCMR registry would very much like to encourage scientists in the field of imaging to submit a proposal for additional new specific protocols to the next registry steering committee meetings http://www.eurocmr-registry.com, available shortly, or http://www.escardio.org/communities/Working-Groups/eurocmr/Pages/welcome.aspx).

### Variables and definitions

**Figure 1:**
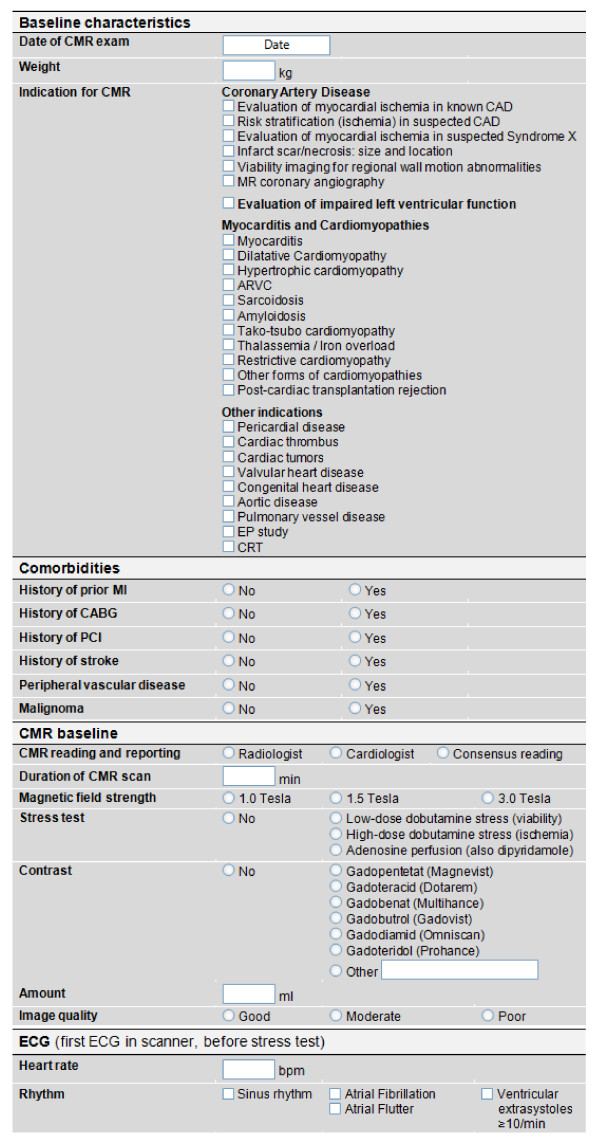
**Baseline characteristics part one - Electronic case record forms to assess baseline characteristics of all enrolled patients and CMR procedural parameters**.

**Figure 2:**
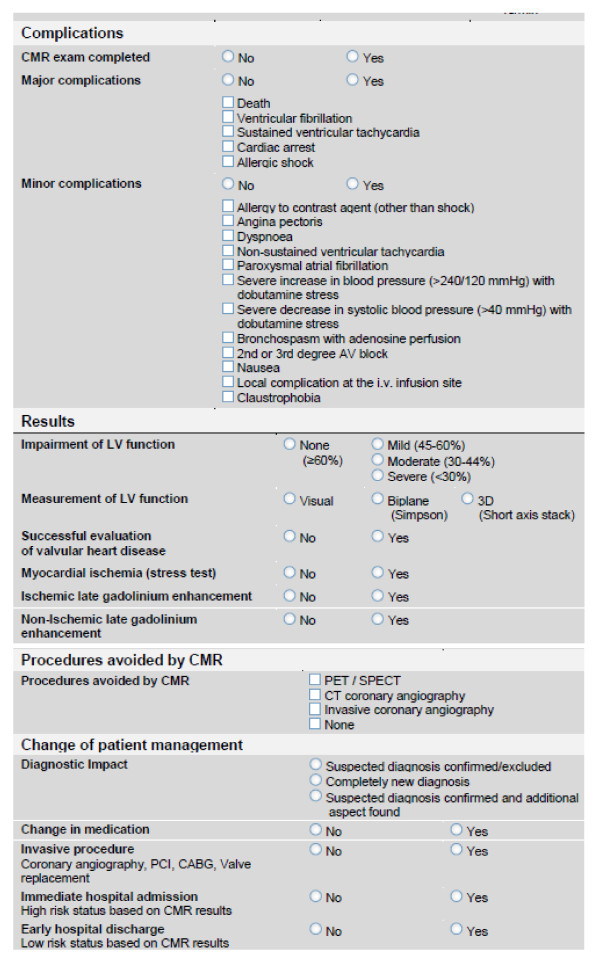
**Baseline characteristics part two - Electronic case record forms to report the results, complications and planed procedures after the CMR scan**.

Electronic case record forms will be used. All variables assessed in the electronic case record form are pre-defined, and will be collected directly from the patients, and/or from the medical records. Variables include demographic data, patient's history, indication for CMR, CMR procedural parameter, results of CMR, complications, and the impact of the CMR procedure on the patient's clinical management (figures 1 and 2). Most fields are self-explanatory; all other fields are defined in the appendix of this manuscript, and are in agreement with recently published key data elements and definitions for cardiac imaging [5].

In order to assess the clinical utility of routine CMR we will document 1) image quality, 2) safety of the procedure and 3) the impact on patient management.

1) To address image quality, studies will be graded in non-diagnostic, moderate but diagnostic, and good quality. A non-diagnostic study does not allow to answer the question the CMR was ordered for. A study will be graded as moderate if the images allow to answer the question the CMR was ordered for, but findings remain questionable due to artefacts. Studies are graded as good if the images are of good quality without artefacts allowing to provide a complete answer to the question the CMR was ordered for.

2) In order to report the safety of CMR in clinical routine, complications of the procedure will be documented. Complications of CMR are defined as severe complications in the setting of death, resuscitation, or any other condition related to the CMR procedure that required monitoring as an inpatient for at least one night after the CMR scan. Mild complications are defined as any complications related to the CMR study not fulfilling the criteria for severe complication.

3) In order to evaluate the impact of CMR on patient management, it will be reported if the CMR scan directly results in a new diagnosis or initiates a direct therapeutic consequence, such as a change in medication, ordering an invasive procedure, hospital admission or discharge.

**Figure 3:**
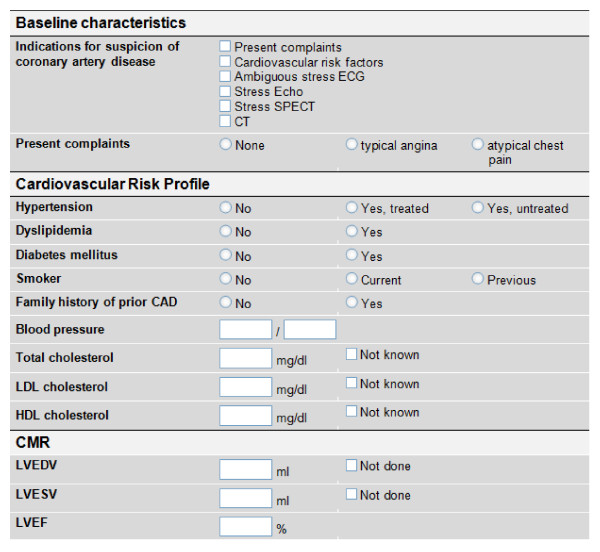
**Specific protocol #1: Suspected-CAD - Electronic case record forms to document the cardiovascular risk profile for patients enrolled in the specific protocol "Suspected CAD"**.

**Figure 4:**
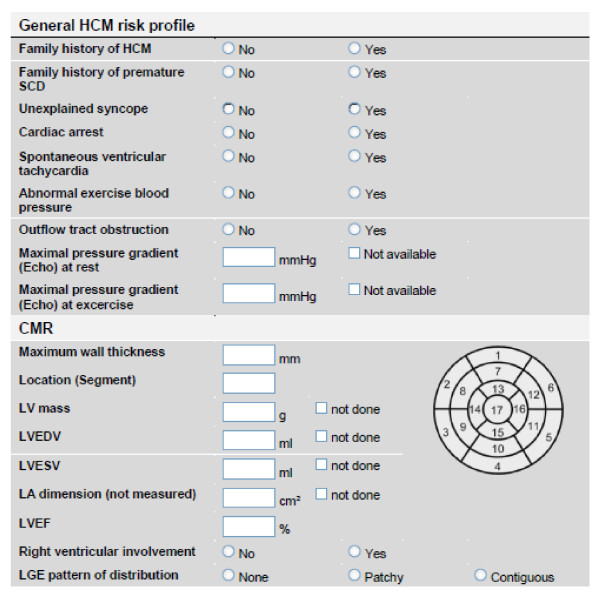
**Specific protocol #2: HCM-SCD - Electronic case record forms to determine the traditional risk factors for sudden cardiac death and CMR findings in patients with HCM enrolled in the specific protocol specific protocol "HCM-SCD"**.

Additional information will be collected for the patients included in the specific protocols. The cardiovascular risk profile for patients enrolled in the specific protocol "Suspected CAD" will be assessed as shown in figure 3. Wall motion and scar burden will be read in a 17-segment model [6]. For patients included in the specific protocol "HCM-SCD" traditional risk factors for sudden cardiac death in patients with HCM will be reported according to figure 4. Wall motion and scar burden will be read in a 17-segment model [6].

### Follow up

**Figure 5:**
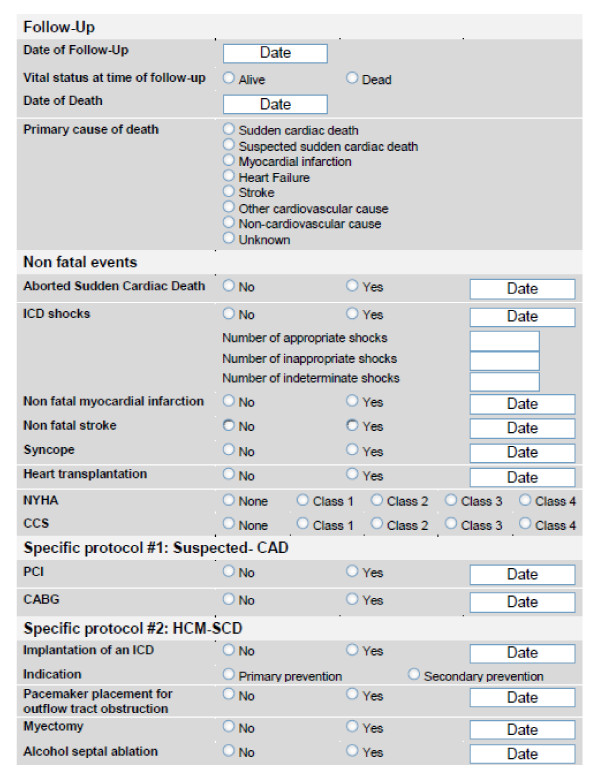
**Follow up - Electronic case record forms to study fatal endpoints, non-fatal endpoints as well as procedures after enrolment for all patients included in specific protocols ("Suspected-CAD" and "HCM-SCD")**.

A clinical follow up will be performed and/or coordinated by the "Institut für Herzinfarktforschung" for all patients enrolled in specific protocols (Suspected-CAD, HCM-SCD) every 12 months after enrolment. Follow ups will be done by standardized telephone interviews. In case of no contact, government registration offices will be asked for new addresses of patient, or cases of death. In non-German speaking countries one local key person appointed by the steering committee that may assist the institute performing the follow up. The endpoint that occurs first is included in the analysis. Fatal endpoints, non-fatal endpoints, as well as procedures after enrolment will be assessed (figure 5).

### Data management

All data will be prospectively collected by trained personnel, manually entered in online case record forms based on database elements provided by the "Institut für Herzinfarktforschung", University of Heidelberg, Germany http://www.herzinfarktforschung.de/Projekte/RegisterI/EuroCMR.html via a SSL-secured internet connection, and stored on a central server. Each participating centre will appoint a senior cardiologist or radiologist as local investigator responsible for the data quality of each individual patient entered in the registry. The local investigator has either to be SCMR level 3 trained, EuroCMR certified, or licensed for CMR by the local chamber of physicians. A plausibility check will directly carried out after submitting the data to avoid further queries as far as possible. For quality control purposes benchmarking reports will be made available to the local investigators of all participating centres on a regular basis. The reports will be compiled individually for each participating centre comparing all data of the centre with those of all other centres. Data collection and management will be approved by local ethics committees.

### Benchmarking reports

Benchmarking reports will be made available to the participating centres on a regular basis. The report will be compiled individually for each participating centre and compares the data of the centre with those of the other centres. The collected data will be evaluated in regard of characteristics, therapy and clinical course of the included patients.

### Quality control

All participating centres have to provide the complete CMR studies of 10% of their patients included selected at random by the central database server. Centres may either upload DICOM images to the central image storage system (WEBPAX, HeartIt, Durham, USA), or sent DICOM CD's to the institute. Members of the steering committee will evaluate all provided cases in consensus read. If necessary, participating centres will be visited by members of the steering committee to address potential quality problems. In addition, a sample of participating centres (approximately 10 sites per year) will be randomly selected for monitoring including source data verification, as well as the completeness of written informed consents.

### Statistics

Since the objectives of this registry are descriptive in nature, no formal hypothesis testing will be done. Absolute numbers and percentages will be computed to describe the patient population. Medians (with quartiles) or means (with standard deviation) will be computed as appropriate. Categorical values will be compared by chi-square test and continuous variables will be compared by two-tailed Wilcoxon rank sum test. P-values < 0.05 were considered significant. All p-values will be results of two-tailed tests. The tests will be performed using the SAS^© ^statistical package, version 9.1 (SAS, Cary, North Carolina). In the specific protocol "Suspected-CAD" the event rates and corresponding 95% confidence intervals (CI) during follow up will be estimated for prespecified subgroups defined by result of CMR. Additionally regression models will be assumed to identify independent risk factors and for calculation of odds ratios. In the specific protocol "HCM-SCD" the event rates and corresponding 95% confidence intervals (CI) during follow up will be estimated for prespecified subgroups defined by detected scar burden. Additionally regression models will be assumed to evaluate CMR parameters such as scar burden as independent risk factors.

Assuming a mortality rate of 3% during the first year after CMR in both specific protocols and a target accuracy (range of CI) lower than 2% of the estimated event rate, the sample size must exceed 1,120 patients in both protocols. This assures that the accuracy of subgroups representing 25% of all patients in both specific protocols will be lower than 4%. In order to assure an accuracy lower than 2% (instead of 4%), the sample size would need to exceed 4,450 patients.

## Discussion

The EuroCMR Registry is the first dataset to assess the clinical utility of routine CMR in a large number of cases in an interdisciplinary multi-centre and multi-vendor setting. The registry will provide the unique opportunity to acquire data on the clinical utility, impact and safety of CMR studies performed in the daily clinical routine. The EuroCMR Registry will give us an insight into the clinical performance of CMR in a broad spectrum of practices of varying size and among operators of varying experience.

In addition, the EuroCMR Registry will evaluate if CMR is able to provide prognostic information by focusing on two specific patient groups, suspected CAD and HCM. The confirmation of improved outcomes attributable to cardiovascular imaging is particularly challenging because of the complex interplay of patient characteristics and treatment strategies, including the substantial variability in therapeutic care patterns after cardiac imaging, and therefore requires a large number of subjects.

A registry offers the advantage to collect data prospectively in a real-life scenario as opposed to a randomized controlled trial. However, it is important to point out that the EuroCMR Registry is subject to the intrinsic limitations of every registry, i.e. the data quality is dependent upon the completeness of reporting and accuracy of the data.

In the future it will be important to assess quality, and further improve quality of CMR imaging in clinical routine. In the recent years there has been increasing concern that less attention has been focused on quality in cardiac imaging in general than other areas of cardiovascular medicine [7]. As pointed out by the Past President of the American College of Cardiology, Pamela Douglas, "the striking growth in the use of cardiovascular diagnostic imaging has been accompanied by a frustrating combination of inconsistent usage, lack of rigorous controls, and little measurable impact on outcomes" [8]. Consequently, several authorities have called for efforts to improve quality in imaging. For example, since "ordering the right test for the right patient is an important component of quality in imaging" [8] novel Appropriateness Criteria have been developed by the American College of Cardiology and the European Society of Cardiology for several imaging modalities including CMR [4,9]. The EuroCMR Registry may be a first step to provide insight if CMR is used according to the novel Appropriateness Criteria. In the future it will be important to not only evaluate if CMR can predict outcome, but also to assess if undergoing CMR and a subsequent change in patient management based on the study findings has any measurable impact on outcome.

## Conclusion

The EuroCMR Registry offers the opportunity to assess the clinical utility and to define the future role of CMR. The EuroCMR Registry also may serve as a tool to assess quality and further improve quality of CMR with subsequent better patient care.

## Competing interests

The EuroCMR Registry is supported by unrestricted educational grants from the following companies (in alphabetic order): Medtronic Inc., Minneapolis MN, USA. Novartis International AG, Basel, Switzerland. and Siemens AG, Medical Solutions, Erlangen, Germany.

## Acknowledgments

The EuroCMR Registry is an official scientific activity of the ESC working group Cardiovascular MR.

## Authors' contributions

AW, OB, HM and EN contributed to the design oft the study, all definitions, and wrote the report. All other authors contributed to the design of the study, reviewed the definitions, as well as the manuscript draft at several stages. This study is a project of the ESC working group "Cardiovascular MR".

## Appendix

List of definitions used in the EuroCMR Registry

• Heart rate: heart rate measured during CMR scan.

• Ventricular extrasystole: includes ventricular extrasystoles during CMR image acquisition.

• Cardiac arrest: abrupt cessation of normal circulation of the blood.

• Allergic shock: serious allergic reaction that is rapid in onset (over minutes to 24 hours after injection of contrast agent), involving the skin, mucosal tissue, or both and evidence of respiratory compromise or reduced blood pressure (systolic BP of less than 90 mmHg or greater than 30% decrease from that person's baseline) or persistent gastrointestinal symptoms.

• Allergy to contrast agent: acute onset of an illness (over minutes to 24 hours after injection of contrast agent) involving the skin, mucosal tissue, or both (eg, generalized hives, pruritus or flushing).

• Non-sustained ventricular tachycardia: monomorphic or polymorphic tachycardia with regard to QRS or electrocardiographic polarity, amplitude, and morphology, with a mean cycle length of less than 240 msec.

• Ventricular fibrillation: Rapid, more than 300 bpm/200 ms (cycle length 180 ms or less), grossly irregular ventricular rhythm with marked variability in QRS cycle length, morphology and amplitude.

• Ventricular flutter: A regular (cycle length variability 30 ms or less) ventricular arrhythmia approximately 300 bpm (cycle length -200 ms) with a monomorphic appearance; no isoelectric interval between QRS complexes.

• Local complications at the iv infusion site include local swelling and/or erythema and prolonged pain at injection site.

• Hypertension: defined as a blood pressure >140/90 mmHg for patients without diabetes or chronic kidney disease, or prior documentation of blood pressure >130/80 mmHg on at least two occasions in patients with diabetes or chronic kidney disease.

• Controlled hypertension: Patient takes currently antihypertensive agent(s) and blood pressure is at goal.

• Uncontrolled hypertension: Patient takes antihypertensive agent(s) and blood pressure is not at goal.

• Dyslipidemia: patient has a history of dyslipidemia diagnosed and/or treated by a physician. Or: Total cholesterol greater than 200 mg/dl (5.18 mmol/l), or Low-density lipoprotein (LDL) greater than or equal to 130 mg/dl (3.37 mmol/l)or High-density lipoprotein (HDL) less than 40 mg/dl (1.04 mmol/l) in men and less than 50 mg/dl (1.30 mmol/l) in women, or currently on antilipidemic treatment.

• Diabetes mellitus: refers to any history or current diabetes (diagnosed by at least 2 fasting glucose measures >7 mmol/L or >126 mg/dl), or a elevated non-fasting glucose level, treated or not.

• Smoker: this includes cigarettes, cigar, tobacco chew, etc. No: subject has never smoked. Current: use of tobacco within 1 month of this study. Previous: use of tobacco greater than 6 months prior to this study.

• Family history of prior CAD: any first-degree relatives (parents, siblings, children) who have had any of the following at age less than 55 years: 1. angina, 2. myocardial infarction (MI), 3. coronary artery bypass graft (CABG), 4. percutaneous coronary intervention (PCI), or 5. sudden cardiac death without obvious cause.

• Blood pressure: systolic and diastolic blood pressure should be measured in a seated position after at least 5 minutes of rest and at the date the CMR is performed.

• Total cholesterol: most recent cholesterol measurement (mg/dl) in medical record up to 6 months prior to CMR study.

• LDL cholesterol: most recent LDL measurement (mg/dl) in medical record up to 6 months prior to CMR study.

• HDL cholesterol: most recent HDL measurement (mg/dl) in medical record up to 6 months prior to CMR study.

• Family history of HCM: any first-degree relatives (parents, siblings, children) who was diagnosed with HCM

• Family history of premature sudden cardiac death: unexpected arrest of presumed cardiac origin that could be interpreted as being cardiac in origin in any first-degree relatives (parents, siblings, children).

• Unexplained syncope: one or more episodes of unexplained loss of consciousness

• Spontaneous ventricular tachycardia: regular (monomorphic) or irregular (polymorphic) tachycardia with regard to QRS or electrocardiographic polarity, amplitude, and morphology, with a mean cycle length of more than 240 msec

• Abnormal exercise blood pressure: systolic blood pressure that fails to increase >20 mm HG during maximal treadmill exercise.

• Outflow tract obstruction: resting peak outflow tract gradient > 30 mmHg by echo.

• Maximal pressure gradient (echo) at exercise: highest gradient measure by pulsed Doppler studies.

• Maximal wall thickness: end-diastolic thickness of the thickest wall measured in a true cross sectional view in the 17 segment model.

• LA dimension: dimension measured in the parasternal long-axis view (4 chamber) similar to echo.

• Fatal endpoints:

1. Sudden cardiac death: unexpected arrest of presumed cardiac origin within one hour after onset of any symptoms that could be interpreted as being cardiac in origin (e.g. death after 30 minutes of angina). If arrest occurs between 1 and 24 hours after onset of symptoms, classify as 'possible' sudden death.

2. Unwitnessed death: subject found deceased with no history of new symptoms within the previous 72 hours.

3. Death within 28 days of cardiac arrest.

4. Death from myocardial infarction: death occurring <28 days after MI.

5. Death from congestive heart failure: documented by the presence of signs of either right or left ventricular failure or both on physical exam or radiographic exam. The diagnosis should be confirmed by non-invasive or hemodynamic measurements.

6. Death from stroke: neurologic symptoms compatible with stroke confirmed by a neurologist prior to death. Or a focal neurologic defect that persists ≥ 24 hours and is confirmed by either a neurologist or by an imaging procedure prior to death. Or confirmed by autopsy.

7. Death from other cardiovascular cause: include aortic dissection, pulmonary embolism, death from non-cardiovascular cause and death from unknown cause.

• Non-fatal endpoints:

1. Aborted sudden cardiac death: resuscitation after cardiac arrest defined as performance of the physical act of cardioversion or CPR in a patient who remains alive 28 days later.

2. ICD shock: Stored data will be analyzed to classify the arrhythmias responsible for precipitating defibrillator discharges, according to the following definitions:

a) Nonsustained tachycardia: see definition above.

b) Nonsustained ventricular tachyarrhythmias that do not activate device therapy are to be excluded.

c) Appropriate ICD shocks: defibrillator discharges that will be considered appropriate include automatic defibrillation shocks or programmed antitachycardia overdrive pacing triggered by ventricular tachycardia or fibrillation and documented by stored intracardiac electrocardiographic or cycle-length data.

d) For events occurring in patients who have defibrillators without the capacity to store electrocardiographic data, discharges will be judged to be appropriate on the basis of clinical findings that strongly suggested the presence of ventricular arrhythmia (i.e., symptoms such as presyncope or syncope immediately before the discharge and the absence of these symptoms immediately afterward).

e) Inappropriate ICD shocks: discharges triggered by a rapid ventricular rate exceeding the programmed threshold rate as a consequence of supraventricular tachycardia, exercise-related sinus tachycardia, or a malfunction of the device.

f) For patients with defibrillators that do not store electrocardiographic data, discharges will be defined as inappropriate if they are not preceded by symptoms, if circumstances suggested the presence of sinus tachycardia due to emotional or physical stress, or if there was a malfunction of the device.

g) Indeterminate ICD shocks: in patients where the there is insufficient data to determine whether an ICD shock is or is not appropriate, the ICD shock will be coded as indeterminate.

3. Non fatal myocardial infarction: typical rise and fall of troponin or CK-MB biomarkers of myocardial necrosis with at least one of the following: ischemic symptoms, development of pathologic Q-waves on the ECG or ECG changes indicative of ischemia (ST elevation or depression) with angiographic evidence of CAD.

4. Non fatal stroke: neurologic symptoms compatible with the diagnosis of stroke as confirmed by neurologist or a focal neurologic defect that persists for ≥ 24 hours and is confirmed by a neurologist or an imaging study.

5. Syncope: one or more episodes of unexplained loss of consciousness.

• NYHA:

1. Class I: patients with cardiac disease but without resulting limitations of physical activity. Ordinary physical activity does not cause undue fatigue, palpitation, or dyspnea.

2. Class II: patients with cardiac disease resulting in slight limitation of physical activity. They are comfortable at rest. Ordinary physical activity results in fatigue, palpitation, or dyspnea.

3. Class III: patients with cardiac disease resulting in marked limitation of physical activity. They are comfortable at rest. Less than ordinary activity causes fatigue, palpitation, or dyspnea.

4. Class IV: patients with cardiac disease resulting in inability to carry on any physical activity without discomfort. Symptoms are present even at rest or minimal exertion.

• CCS:

1. Class 0: Asymptomatic. No angina.

2. Class I: Ordinary physical activity (e.g., walking or climbing stairs) does not cause angina; angina occurs with strenuous or rapid or prolonged exertion at work or recreation.

3. Class II: Slight limitation of ordinary activity (e.g., angina occurs walking or stair climbing after meals, in cold, in wind, under emotional stress, or only during the few hours after awakening; walking more than 2 blocks on the level or climbing more than 1 flight of ordinary stairs at a normal pace; and in normal conditions).

4. Class III: Marked limitation of ordinary activity (e.g., angina occurs with walking 1 or 2 blocks on the level or climbing 1 flight of stairs in normal conditions and at a normal pace).

5. Class IV: Inability to perform any physical activity without discomfort; angina syndrome may be present at rest.

• Procedures following enrolment:

1. Protocol "Suspected-CAD": percutaneous coronary intervention (PCI) and coronary artery bypass grafting (CABG), first performed procedure after CMR, successful or not.

2. Protocol "HCM-SCD": ICD placement for primary or secondary prevention, pacemaker placement for outflow tract obstruction, myectomy (with or without another procedure), and alcohol septal ablation.
